# Outcomes of Technical Variant Liver Transplantation versus Whole Liver Transplantation for Pediatric Patients: A Meta-Analysis

**DOI:** 10.1371/journal.pone.0138202

**Published:** 2015-09-14

**Authors:** Hui Ye, Qiang Zhao, Yufang Wang, Dongping Wang, Zhouying Zheng, Paul Michael Schroder, Yao Lu, Yuan Kong, Wenhua Liang, Yushu Shang, Zhiyong Guo, Xiaoshun He

**Affiliations:** 1 Organ Transplant Center, The First Affiliated Hospital, Sun Yat-sen University, Guangzhou 510080, China; 2 University of Toledo College of Medicine, Toledo, Ohio 43614, United States of America; Penn State University, UNITED STATES

## Abstract

**Objective:**

To overcome the shortage of appropriate-sized whole liver grafts for children, technical variant liver transplantation has been practiced for decades. We perform a meta-analysis to compare the survival rates and incidence of surgical complications between pediatric whole liver transplantation and technical variant liver transplantation.

**Methods:**

To identify relevant studies up to January 2014, we searched PubMed/Medline, Embase, and Cochrane library databases. The primary outcomes measured were patient and graft survival rates, and the secondary outcomes were the incidence of surgical complications. The outcomes were pooled using a fixed-effects model or random-effects model.

**Results:**

The one-year, three-year, five-year patient survival rates and one-year, three-year graft survival rates were significantly higher in whole liver transplantation than technical variant liver transplantation (OR = 1.62, 1.90, 1.65, 1.78, and 1.62, respectively, *p*<0.05). There was no significant difference in five-year graft survival rate between the two groups (OR = 1.47, *p* = 0.10). The incidence of portal vein thrombosis and biliary complications were significantly lower in the whole liver transplantation group (OR = 0.45 and 0.42, both *p*<0.05). The incidence of hepatic artery thrombosis was comparable between the two groups (OR = 1.21, *p* = 0.61).

**Conclusions:**

Pediatric whole liver transplantation is associated with better outcomes than technical variant liver transplantation. Continuing efforts should be made to minimize surgical complications to improve the outcomes of technical variant liver transplantation.

## Introduction

By the 1980s, pediatric liver transplantation had become the standard treatment for infants or children suffering from life-threatening end-stage liver disease [1]. Up to the early 1980s, the only technical option for pediatric liver transplantation was to transplant a whole liver graft with a weight as close as possible to that of the recipient [2]. More than 50% of the children on the waiting list would die before they could receive a transplant in the 1980s [3]. The technical variant techniques, including reduced-size (RSL)[4], split (SLT)[5], and living donor liver transplantation (LLT)[6], can use a partial liver graft to replace the role of a whole organ, thereby releasing the burden of appropriate-sized whole liver grafts.

Nowadays, pediatric liver transplantation has become one of the most successful solid organ transplantations, with a five-year patient survival rate exceeding 70% [7]. And there are some well-known factors influencing the early survival rate, including age [8–10], primary diseases [11–14], and severity of illness [15]. However, the impact of graft types (whole, reduced, split, or living-donor) on transplant outcomes is less clear. Herein, we perform a meta-analysis of studies concerning pediatric liver transplants to compare patient/graft survival and incidence of surgical complications between whole liver transplantation (WLT) and technical variant liver transplantation (TVLT).

## Methods

### Study Design and Search Strategy

Before data collection, two liver transplantation groups were identified for comparison: WLT and TVLT. TVLT included three subgroups, namely RLT, SLT, and LLT. We also compared these individual subgroups to WLT.

To identify relevant studies up to January 2014, we searched PubMed/Medline, Embase, and Cochrane library databases. In addition, reference lists were scanned to identify additional potentially relevant studies. The search strategy included the terms: pediatric or children or infant, liver transplantation, and technical variant grafts or reduced or split or living donor liver transplantation. We included trials with no language or year restrictions.

### Inclusion and Exclusion Criteria

This meta-analysis only included studies meeting the following criteria: (1) studies were conducted in pediatric patients; and (2) studies were aimed to compare patient and graft survival rates, and incidence of complications between WLT and TVLT. Studies were excluded when they met the following criteria: (1) studies reporting combined organ transplantation; and (2) case reports, conference abstracts, or journal editorials.

### Data Extraction

For the trials included in our meta-analysis, we sought data for authors, journal and year of publication, number of patients, demographic information, patient and graft survival rates, and incidences of surgical complication. The primary outcomes of the current meta-analysis were patient and graft survival rates, and the secondary outcomes were the incidence of surgical complications. When the data were not shown in the article, we attempted to contact the authors to obtain original information. The data were extracted by two investigators (YW and YK) independently, and disagreements were resolved by intervention of a third author (ZG). The conduct and reporting were in accordance with the Quality of Reporting of Meta-Analyses statement.

### Statistical Analysis

We used the statistical software Review Manager 5.1 (The Cochrane Collaboration, Oxford, United Kingdom) to analyze the collected data. The primary outcomes and the secondary outcomes were analyzed as dichotomized variables, and their results were reported as pooled odds ratio (OR) with 95% confidence interval (CI). Cumulative meta-analyses of the primary and secondary outcomes between WLT and TVLT were also carried out, to assess the evolution of the outcomes in time. The heterogeneity of included trials was measured by heterogeneity index *I* ^*2*^. We used an *I* ^*2*^ value of 50% as the line that is between heterogeneity and homogeneity in our meta-analysis [16]. And a *p* value of <0.05 indicates significant heterogeneity. When the result showed homogeneity, a fixed-effects model was used to estimate the pooled effect on outcomes. When results showed heterogeneity, we use a random-effects model.

### Assessment of Publication Bias

Graphical funnel plots were generated so that we could make visual inspections for publication bias [17]. The statistical methods used for detecting funnel plot asymmetry were the Egger’s regression asymmetry test [17] and Begg-Mazumdar rank correlation test [18].

## Results

### Study Characteristics

**Fig 1:**
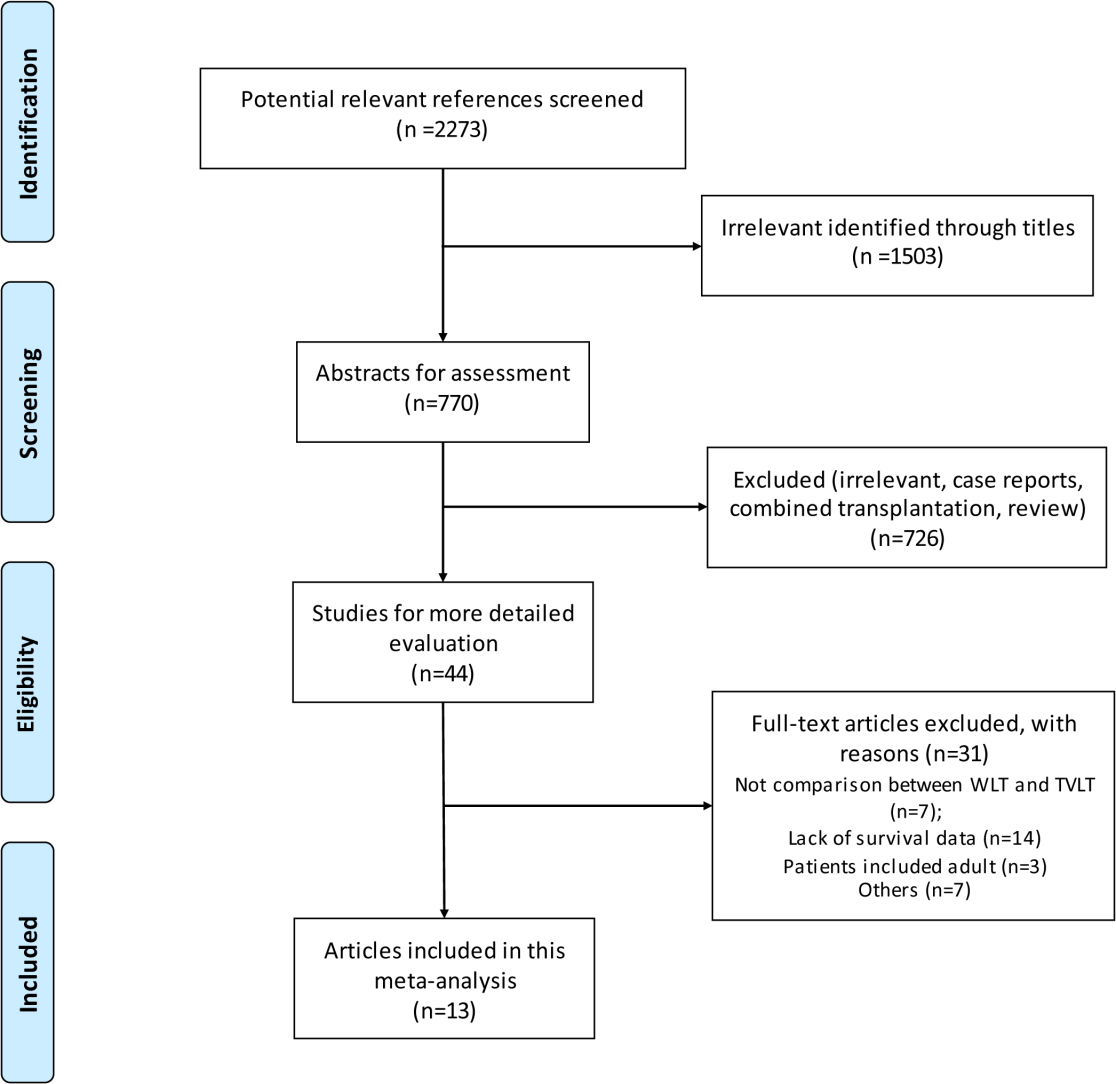
Flow diagram of study identification.

We screened 2273 citations, from which we identified 13 articles to assess patient/graft survival rate as well as incidence of surgical complications between WLT and TVLT. Details on the study selection process are presented in Fig 1. All the studies included in our meta-analysis were observational studies.

The 13 articles [19–31] were published from 1994 to 2010 and included 3662 pediatric patients and 3801 transplantations. The studies were from nine countries, including the United States (3), Spain (3), France (1), Italy (1), Belgium (1), Brazil (1), Netherlands (1), Sweden (1) and Japan (1). The basic characteristics of the included studies are showed in Table 1.

**Table 1:** Basic characteristics of the studies included in this meta-analysis.

Study	Year	Country	Study design	Number of Patients	Number of transplants				Median age (months)(WLT/TVLT)	Body weight (kg)(WLT/TVLT)
					WLT	RLT	SLT	LLT		
**Asensio [19]**	2001	Spain	Cohort study	100	102	27	0	0	70.8/38.1	20.5/11.9
**Burgos [20]**	2008	Spain	Cohort study	103	25	32	17	29	98.8/40.7	29.6/14.0
**Cacciarelli [21]**	1997	USA	Cohort study	101	49	68	0	0	<23*	NA
**Chardot [22]**	1999	France	Cohort study	179	76	112	16	0	33.0/28.0	12.6/12.0
**D'Alessandro [23]**	2007	USA	Cohort study	155	128	50	18	0	61.6†	20.6†
**Diamond [24]**	2007	USA	Cohort study	2192	1183	388	261	360	79.2/35.8	NA
**Gridelli [25]**	2003	Italy	Cohort study	124	30	8	100	0	13.2†	8†
**Leal [26]**	2007	Spain	Cohort study	83	24	37	8	14	<12*	NA
**Otte [27]**	1998	Belgium	Cohort study	416	168	174	21	53	<15*	NA
**Salzedas [28]**	2010	Brazil	Cohort study	41	20	5	17	0	115/43.3	19.8/9.7
**Sieders [29]**	1999	Netherlands	Cohort study	97	47	0	50	0	54.0/22.8	15.5/10.3
**Suwata [30]**	1994	Sweden	Cohort study	30	14	19	0	0	63.6/64.8	25.4/19.4
**Yamauchi [31]**	2006	Japan	Cohort study	41	17	27	0	7	72.0/36.0	NA

*.The details of patients age were unknown

†.The median age of total patients in each study

Abbreviation: NA, not available; WLT, whole liver transplantation; RLT, reduced-size liver transplantation; SLT, split liver transplantation; LLT, living donor liver transplantation.

### Patient Survival

#### One-year patient survival rate

**Fig 2:**
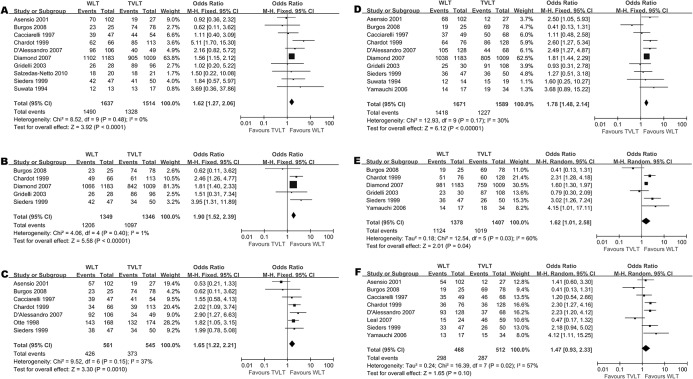
Meta-analysis of survival rate between WLT and TVLT: (A) 1-year patient survival, (B) 3-year patient survival, and (C) 5-year patient survival rate between WLT and TVLT; (D) 1-year graft survival, (E) 3-year graft survival, and (F) 5-year graft survival rate between WLT and TVLT.

We conducted a meta-analysis (10 trials with 3151 patients) of one-year patient survival rates between the WLT and TVLT groups. The one-year patient survival rate was higher in recipients undergoing WLT versus TVLT (OR = 1.62 [1.27–2.06], *p* < 0.05), with low heterogeneity (*I* ^*2*^ = 0%, *p* = 0.48) (Fig 2A). Similar results were observed between WLT and RLT (OR = 1.96 [1.47–2.61], *p* < 0.05; *I* ^*2*^ = 18%, *p* = 0.29) (S1A Fig), as well as between WLT and SLT (OR = 1.72 [1.15–2.58], *p* = 0.009; *I* ^*2*^ = 27%, *p* = 0.24) (S1B Fig).

#### Three-year patient survival rate

Recipients receiving WLT had a higher three-year survival rate than those receiving TVLT (OR = 1.90 [1.52–2.39], *p* < 0.05) (Fig 2B). The heterogeneity between studies was not significant (*I* ^*2*^ = 1%, *p* = 0.40). Similar results were documented between the WLT and RLT groups (OR = 2.15 [1.62–2.85], *p* < 0.05; *I* ^*2*^ = 0%, *p* = 0.52) (S1C Fig). However, there was no statistical difference between the WLT and SLT groups (OR = 2.55 [0.66–9.87], *p* = 0.17; *I* ^*2*^ = 69%, *p* = 0.02) (S1D Fig).

#### Five-year patient survival rate

The five-year patient survival rate was higher in the WLT group versus the TVLT group (OR = 1.65 [1.22–2.21], *p* = 0.001; *I* ^*2*^ = 37%, *p* = 0.15) (Fig 2C). The five-year patient survival rates were comparable between WLT and RLT (OR = 1.50 [0.96–2.36], *p* = 0.08; *I* ^*2*^ = 41%, *p* = 0.13) (S1E Fig), as well as between WLT and SLT (OR = 3.06 [0.30, 31.40], *p* = 0.35; *I* ^*2*^ = 61%, *p* = 0.08) (S1F Fig).

### Graft Survival

#### One-year graft survival rate

The one-year graft survival rate was higher in the WLT group than the TVLT group (OR = 1.78 [1.48–2.14], *p* < 0.05; *I* ^*2*^ = 30%, *p* = 0.17) (Fig 2D). Similar results were observed between the WLT and RLT groups (OR = 2.16 [1.72–2.70], *p* < 0.05; *I* ^*2*^ = 20%, *p* = 0.27) (S2A Fig), as well as between the WLT and SLT (OR = 1.57, [1.15–2.14], *p* = 0.004; *I* ^*2*^ = 34%, *p* = 0.19) (S2B Fig).

#### Three-year graft survival rate

The three-year graft survival rate was higher in WLT versus TVLT (OR = 1.62 [1.01–2.58], *p* = 0.04; *I* ^*2*^ = 60%, *p* = 0.03) (Fig 2E). The three-year graft survival rate was also higher in WLT versus RLT (OR = 1.91 [1.51–2.42], *p* < 0.05; *I* ^*2*^ = 32%, *p* = 0.21) (S2C Fig). However, the three-year graft survival rate between WLT and SLT was comparable (OR = 1.54 [0.51–4.66], *p* = 0.45; *I* ^*2*^ = 76%, *p* = 0.006) (S2D Fig).

#### Five-year graft survival rate

The five-year graft survival rate was comparable between WLT and TVLT (OR = 1.47 [0.93–2.33], *p* = 0.10; *I* ^*2*^ = 57%, *p* = 0.02) (Fig 2F), as well as between WLT and SLT (OR = 0.81 [0.12–5.45], *p* = 0.83; *I* ^*2*^ = 71%, *p* = 0.02) (S2F Fig). However, the five-year graft survival rate was higher in WLT versus RLT (OR = 1.63 [0.99–2.70], *p* = 0.05; *I* ^*2*^ = 54%, *p* = 0.04) (S2E Fig).

### Surgical Complications

**Fig 3:**
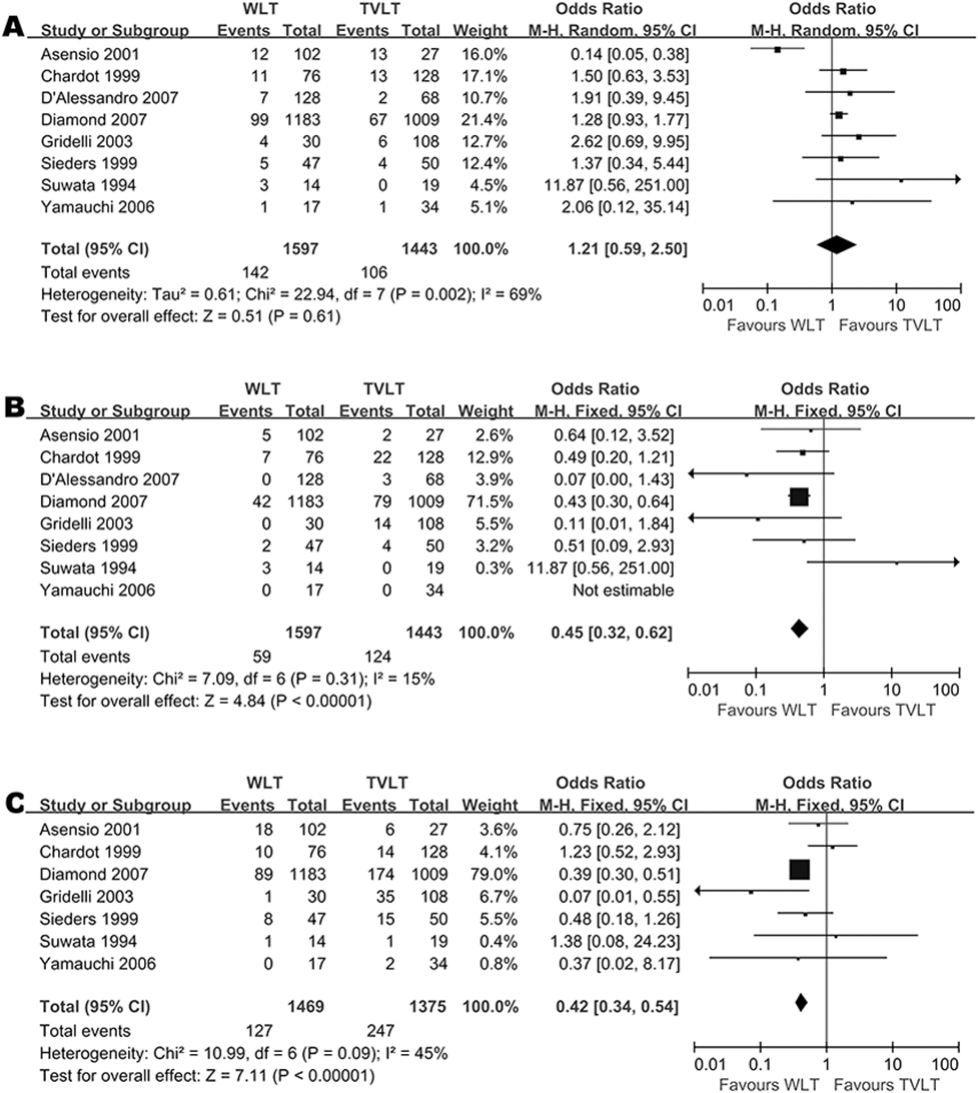
Meta-analysis of complication incidence between WLT and TVLT: The incidence of hepatic artery thrombosis (HAT) (A), portal vein thrombosis (PVT) (B), and biliary complications (BC) (C), in WLT and TVLT.

We selected the most common surgical complications for analysis, namely, hepatic arterial thrombosis (HAT), portal venal thrombosis (PVT) and biliary complications (BC). The incidence of HAT was comparable between WLT and TVLT (OR = 1.21 [0.59–2.50], *p* = 0.61; *I* ^*2*^ = 69%, *p* = 0.002) (Fig 3A). However, the meta-analyses of PVT (OR = 0.45 [0.32–0.62], *p* < 0.05) (Fig 3B) and BC (OR = 0.42 [0.34–0.54], *p* < 0.05) (Fig 3C) between WLT and TVLT showed significant benefits favoring the WLT group with low heterogeneity across studies (*I* ^*2*^ = 15%, *p* = 0.31; *I* ^*2*^ = 45%, *p* = 0.09, respectively). When we compared WLT and RLT, there were no significant differences in the incidence of the three complications (HAT, *p* = 0.06; PVT, *p* = 0.07; and BC, *p* = 0.15) (S3A–S3C Fig). The comparisons between WLT and SLT showed that the incidence of HAT was also comparable between the two groups (*p* = 0.07) (S3D Fig), while PVT and BC occurred more frequently in the SLT group (both *p* < 0.05) (S3E and S3F Fig). The comparison of the incidence of these complications between WLT and LLT could not be performed due to insufficient data.

### Cumulative Meta-analysis

**Fig 4:**
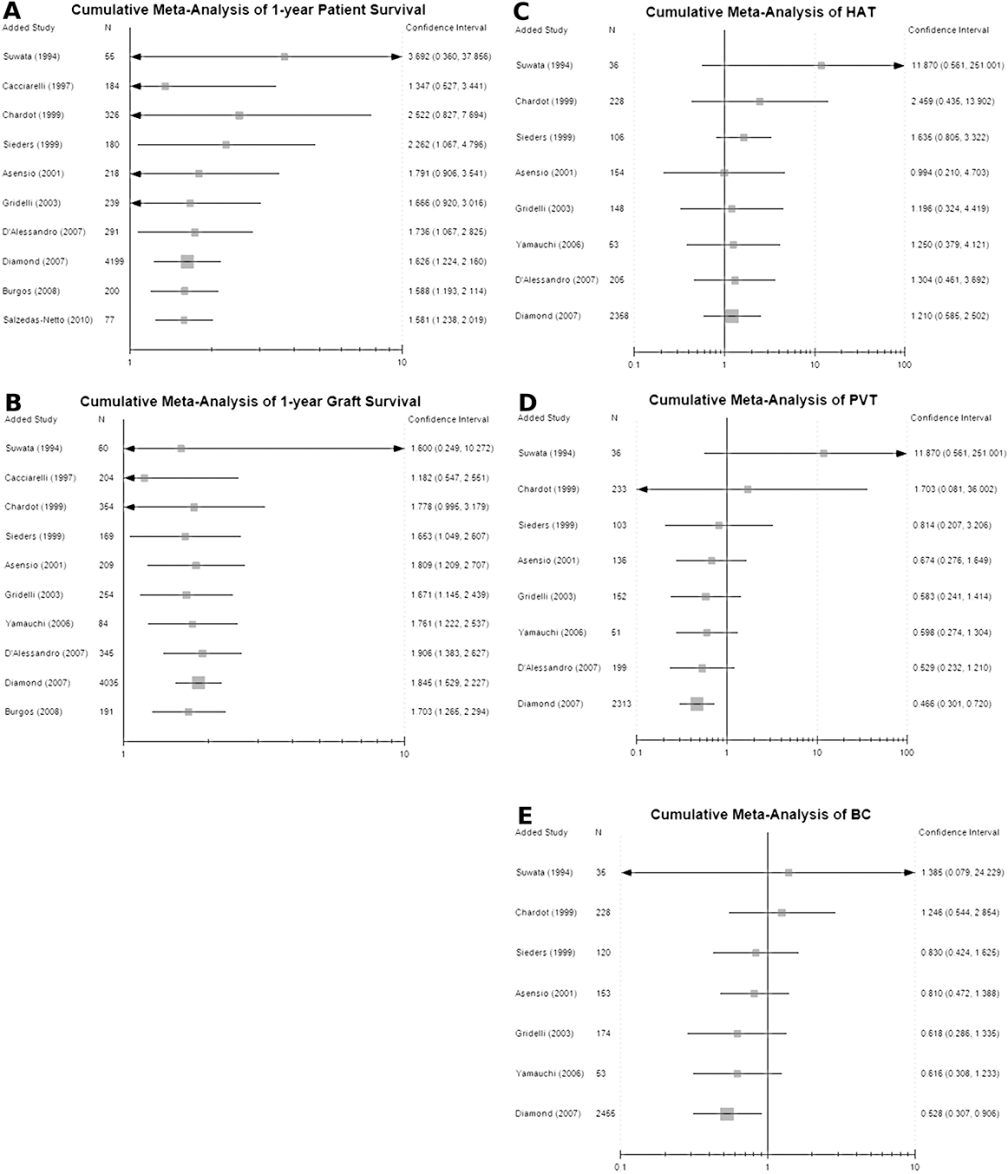
Cumulative meta-analysis of 1-year survival rate and complication incidence between WLT and TVLT: (A) 1-year patient survival rate in WLT and TVLT; (B) 1-year graft survival rate in WLT and TVLT. The incidence of hepatic artery thrombosis (HAT) (C), portal vein thrombosis (PVT) (D), and biliary complications (BC) (E) in WLT and TVLT.

We conducted cumulative meta-analyses of patient survival rate, graft survival rate and complications between WLT and TVLT to evaluate any changes in the outcomes. A cumulative meta-analysis of one-year patient survival rate showed that since 2007 the statistically significant difference between the two groups appeared (Fig 4A), while the cumulative meta-analysis of one-year graft survival rate showed that a statistical difference between the two groups appeared since 1999 (Fig 4B). The cumulative meta-analyses of 3-year and 5-year survival rates are presented in S4 Fig. We also conducted a cumulative meta-analysis of the incidence of complications between WLT and TVLT. The results showed that there was no statistical difference in HAT between WLT and TVLT through all the time, while the CI was getting narrower (OR = 1.21 [0.58–2.50]) (Fig 4C). In contrast, since 2007 statistical differences in PVT and BC appeared between the two groups favoring the WLT group (OR = 0.47 [0.30, 0.72] and 0.53 [0.31, 0.91], respectively) (Fig 4D and 4E).

### Publication bias

The appearance of the funnel plot analysis was symmetrical (S5 Fig), and there was no evidence of publication bias. The *p* values of Begg’s test for one-year, three-year, five-year patient survival rates and one-year, three-year, five-year graft survival rate comparisons between WLT and TVLT were 1.00, 0.81, 0.76, 0.59, 1.00, and 0.54, respectively, meanwhile the Egger’s test showed *p* values of 0.49, 0.46, 0.96, 0.58, 0.52 and 0.60, respectively.

## Discussion

We comprehensively reviewed the existing literature on comparison of survival rates and incidence of complications between pediatric WLT and TVLT. The one-year, three-year, and five-year patient survival rate, as well as one-year and three-year graft survival rate were significantly higher in the WLT group compared to the TVLT group. There was no significant difference in five-year graft survival rate between the two groups. When it comes to complications, the results suggested a lower incidence of PVT and BC in the WLT group. The incidence of HAT was comparable between the groups. In addition, our cumulative meta-analysis showed that the difference in one-year patient survival has been present since 2007 and the difference in one-year graft survival has been present since 1999. Collectively, these results show that WLT is associated with a better outcome when compared to TVLT.

Pediatric and small-sized donor organs are a scarce resource, making it difficult to find adequate grafts for small-sized transplant candidates [32]. Some centers have reported that with the successful application of TVLT, the time on the waitlist and pre-transplantation mortality has been reduced dramatically without compromising patient outcomes [33–40]. An equipoise analysis of the use of RLT demonstrates that a 50% postoperative mortality rate is acceptable and does not increase overall mortality [41]. In addition, according to the latest update in the database of the U.S. Organ Procurement and Transplantation Network and the Scientific Registry of Transplantation Recipients (OPNT/SRTR) [7], the pediatric donors decreased from 878 in 2000 to 790 in 2009, suggesting that the number of pediatric WLT likely decreased through the same period. However, the transplant rates among pediatric patients wait-listed for a liver transplantation increased from 70.9% to 83.1% between 1998 and 2008, and in the same decade both the median waiting time and pre-transplantation mortality in the waiting list have decreased dramatically. These data clearly show that the extensive use of TVLT in pediatric patient has achieved a significant success in expanding the donor pool for children. Although we could not compare the LLT to WLT due to lack of sufficient data, there are four articles based on the UNOS database demonstrating that patients undergoing LLT experience superior survivals compared to patients treated with WLT [42–45]. Therefore, TVLT not only expands the donor pool but also facilitates earlier transplant, providing an increased chance of survival [24]. Therefore, TVLT may be a wiser choice than just waiting when WLT is unavailable. However, more studies, particularly intention-to-treat studies, are needed to provide more evidence to support this point.

Importantly, the comparisons between subgroups (RLT and SLT) of TVLT and WLT demonstrate that the survival difference mainly exists in the short-term outcomes. These results are in accordance with previous studies [45, 46]. Primary nonfunctional, HAT, hepatic venous outflow obstruction and portal venous complications are the top causes of liver graft failure, and infections are the main reason for patient death with functioning graft [47–54]. All of these occur within a short time post-transplantation. Our meta-analysis shows that PVT and BC occurred more frequently in the TVLT group, suggesting a higher incidence of these complications may explain the inferior survival in the TVLT group. Indeed, Backman et al have reported a 3.4 times higher graft loss rate for the first two years compared with three to five years post-transplantation [55]. On the other hand, Buell et al have pointed out that the use of living related grafts with portal vein conduits carries a higher incidence of portal vein complications and reduced-size and split livers are most susceptible to hepatic vein stenosis [35]. That study also confirmed that the use of venous conduits and perioperative vascular thrombosis correlates with a higher incidence of late venous complications. Thus, the innovative surgical techniques, which avoid using these venous conduits, can decrease the incidence of portal vein and biliary complications. In addition, some publications have demonstrated that SLT results in better patient and graft survival when performed in experienced centers [56–58]. These reports suggest that minimization of short-term surgical complications probably help decrease graft loss and patient deaths.

Interestingly, our cumulative meta-analyses show that the short-term (one-year) survival outcomes after transplantation presented a significant trend over years that the difference between WLT and TVLT is getting smaller and smaller chronologically. The three-year survival rates also showed a similar trend. Therefore, the outcome of TVLT is continuously improving and may eventually achieve similar outcomes to that of WLT in the near future. Notably, the cumulative meta-analysis of complications demonstrated that, since 2007, statistical differences in PVT and BC between WLT and TVLT appeared, favoring the WLT group. Although there is no statistically significant difference in HAT incidence between WLT and TVLT, transplantation surgeons should try their best to avoid the occurrence of HAT, since HAT is a common complication and among the major causes for graft loss [48]. These results together, point out that one way to further improve the outcomes in TVLT recipients is to modify the surgical techniques to decrease the incidence of PVT and BC post-transplantation.

To our knowledge, this meta-analysis is the first systematic study of all available data on the comparison between WLT and TVLT in pediatric patients. The 13 analyzed studies come from nine different countries. Therefore, the bias due to racial, diet, environmental, and etiological differences could be minimized. Overall, this study provides the most convincing results on the topic so far. However, undoubtedly, there are some limitations. First, there was no randomized study included, and all included studies were observational studies. Given the reality of organ allocation, surgeons rarely have the luxury of randomizing the graft type. Second, we did not compare the LLT to WLT because of lack of data. Although there were numerous published papers focusing on LLT, they usually made the comparison between living donors and deceased donors, rarely compared LLT to WLT. All these limitations provide room for future studies to provide more information for better choice of graft types.

In conclusion, this study suggests that currently, the pediatric WLT procedure has better outcomes than TVLT procedures. The difference between WLT and TVLT mainly exists in the short-term outcomes. Physicians should weigh the risks and benefits of performing a timely TVLT or waiting for a better WLT. Importantly, continuing efforts should be made to minimize surgical complications to improve the outcomes of TVLT. Multicenter, prospective, match controlled studies may add more information to the findings of this study.

## Supporting Information

S1 PRISMA Checklist The PRISMA 2009 Checklist. (DOC)

S1 Fig The Meta-analysis of patient survival rate between WLT and RLT, and between WLT and SLT. (A) 1-year patient survival rate between WLT and RLT, (B) 1-year patient survival rate between WLT and SLT, (C) 3-year patient survival rate between WLT and RLT, (D) 3-year patient survival rate between WLT and SLT, (E) 5-year patient survival rate between WLT and RLT, and (F) 5-year patient survival rate between WLT and SLT.
(TIF)

S2 Fig The Meta-analysis of graft survival rate between WLT and RLT, and between WLT and SLT. (A) 1-year graft survival rate between WLT and RLT, (B) 1-year graft survival rate between WLT and SLT, (C) 3-year graft survival rate between WLT and RLT, (D) 3-year graft survival rate between WLT and SLT, (E) 5-year graft survival rate between WLT and RLT, (F) 5-year graft survival rate between WLT and SLT.
(TIF)

S3 Fig The Meta-analysis of complication incidence between WLT and RLT, and between WLT and SLT. The incidence of hepatic artery thrombosis (HAT) (A), portal vein thrombosis (PVT) (B), and biliary complications (BC) (C) in WLT and RLT. The incidence of HAT (D), PVT (E), and BC (F) in WLT and SLT.
(TIF)

S4 Fig The Cumulative meta-analysis of 3-year and 5-year patient and graft survival rate between WLT and TVLT. (A) 3-year patient survival rate between WLT and TVLT, (B) 3-year graft survival rate between WLT and TVLT, (C) 5-year patient survival rate between WLT and TVLT, (D) 5-year graft survival rate between WLT and TVLT. PDF.
(TIF)

S5 Fig The Funnel plots for assessing publication bias. (A) 1-year patient survival, (B) 3-year patient survival, and (C) 5-year patient survival between WLT and TVLT; (D) 1-year graft survival, (E) 3-year graft survival, and (F) 5-year graft survival between WLT and TVLT.
(TIF)

S1 Text List of full-text excluded articles. (DOCX)
