# Supplementary figures and images for: Outcomes of Technical Variant Liver Transplantation versus Whole Liver Transplantation for Pediatric Patients: A Meta-Analysis
Source: PLoS One. 2015 Sep 14;10(9):e0138202. doi: 10.1371/journal.pone.0138202 (PMC4569420; doi:10.1371/journal.pone.0138202)

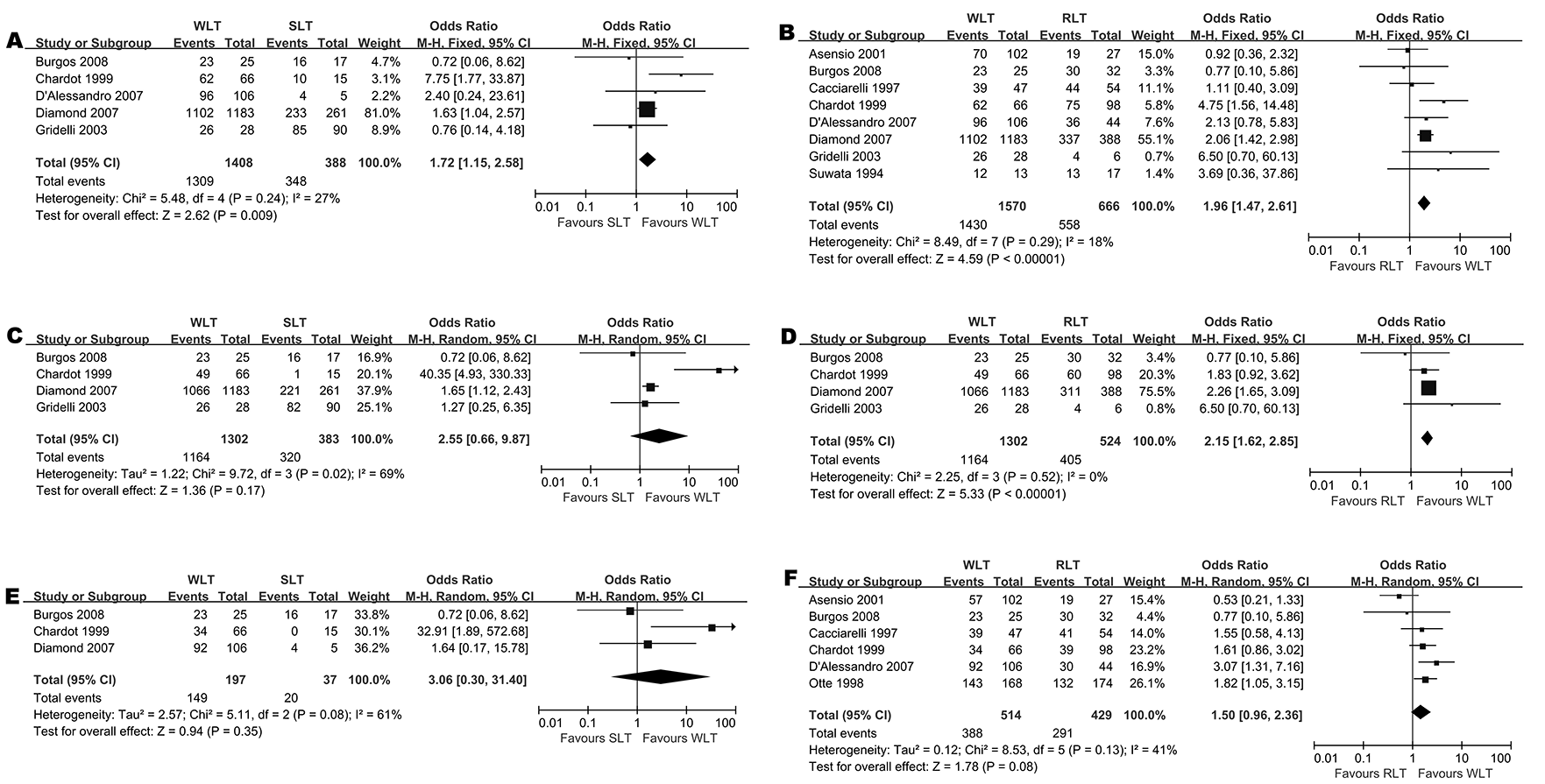

Supplement: S1 Fig — (A) 1-year patient survival rate between WLT and RLT, (B) 1-year patient survival rate between WLT and SLT, (C) 3-year patient survival rate between WLT and RLT, (D) 3-year patient survival rate between WLT and SLT, (E) 5-year patient survival rate between WLT and RLT, and (F) 5-year patient survival rate between WLT and SLT. (TIF) [file pone.0138202.s002.tif]

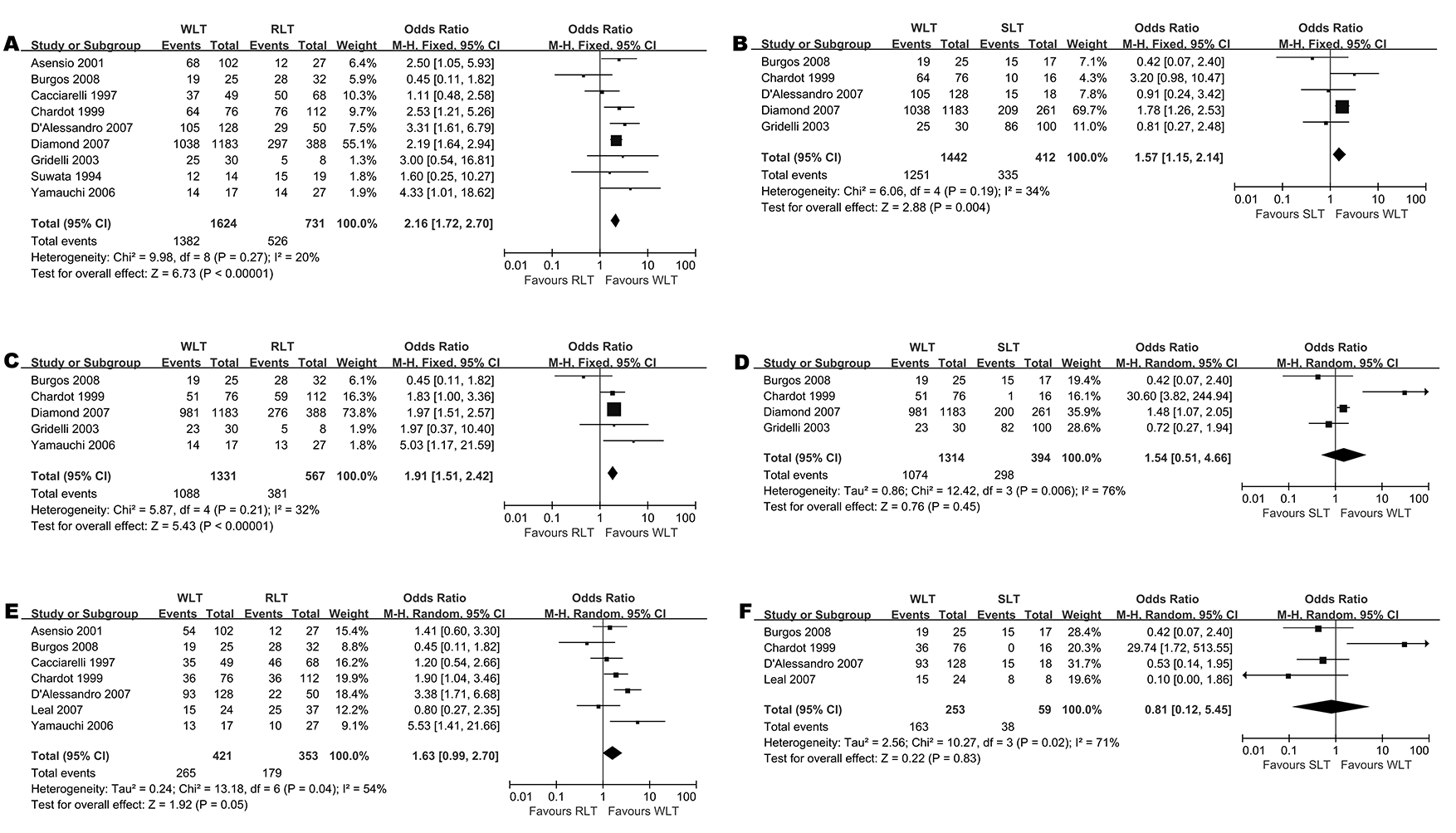

Supplement: S2 Fig — (A) 1-year graft survival rate between WLT and RLT, (B) 1-year graft survival rate between WLT and SLT, (C) 3-year graft survival rate between WLT and RLT, (D) 3-year graft survival rate between WLT and SLT, (E) 5-year graft survival rate between WLT and RLT, (F) 5-year graft survival rate between WLT and SLT. (TIF) [file pone.0138202.s003.tif]

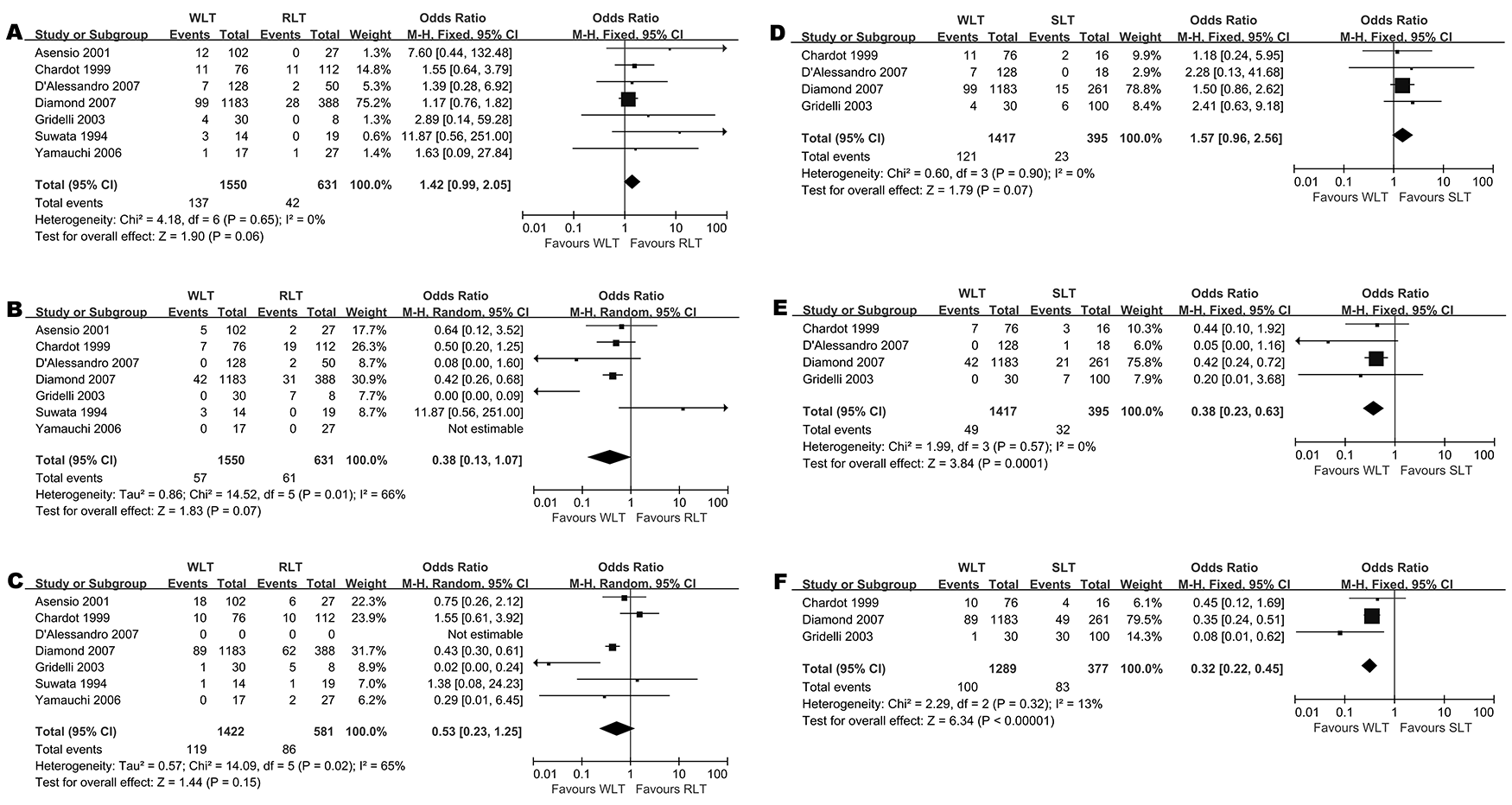

Supplement: S3 Fig — The incidence of hepatic artery thrombosis (HAT) (A), portal vein thrombosis (PVT) (B), and biliary complications (BC) (C) in WLT and RLT. The incidence of HAT (D), PVT (E), and BC (F) in WLT and SLT. (TIF) [file pone.0138202.s004.tif]

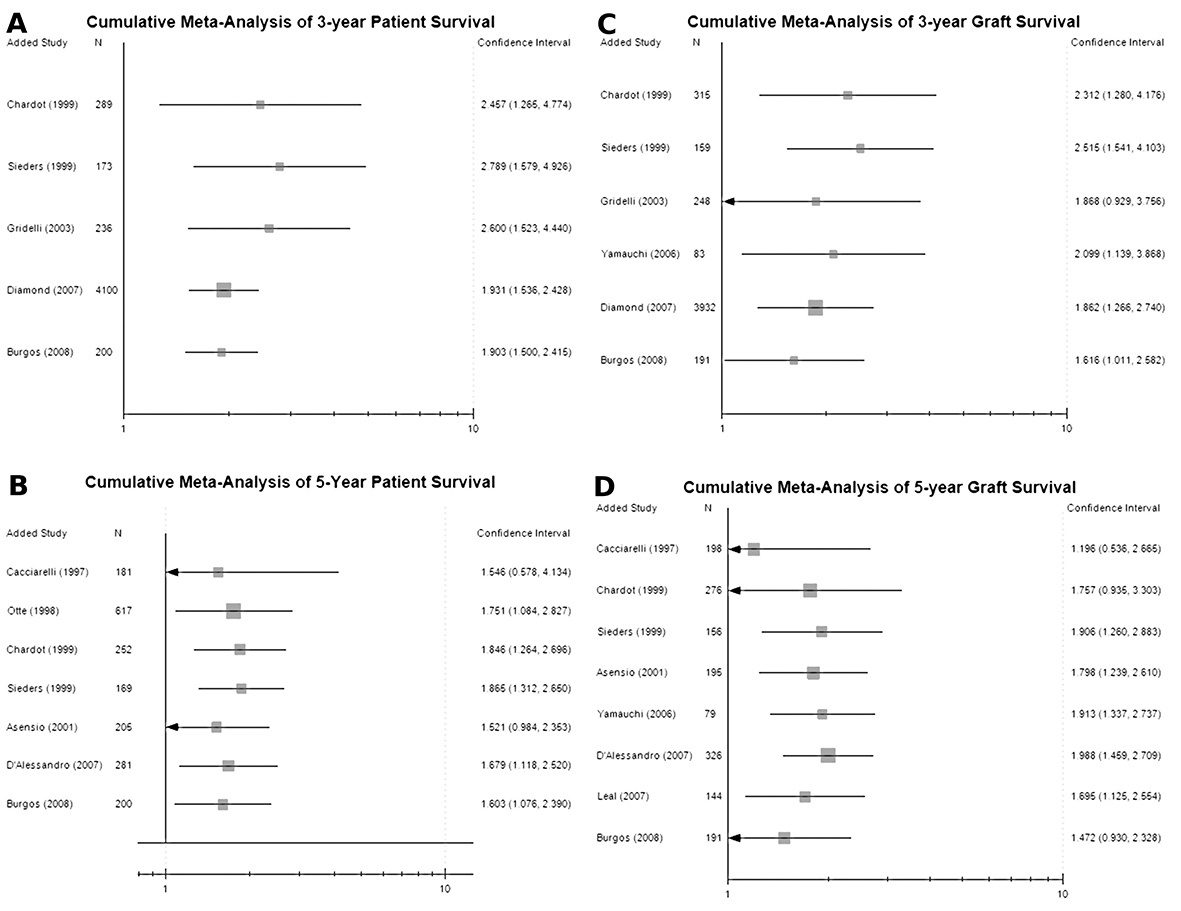

Supplement: S4 Fig — (A) 3-year patient survival rate between WLT and TVLT, (B) 3-year graft survival rate between WLT and TVLT, (C) 5-year patient survival rate between WLT and TVLT, (D) 5-year graft survival rate between WLT and TVLT. PDF. (TIF) [file pone.0138202.s005.tif]

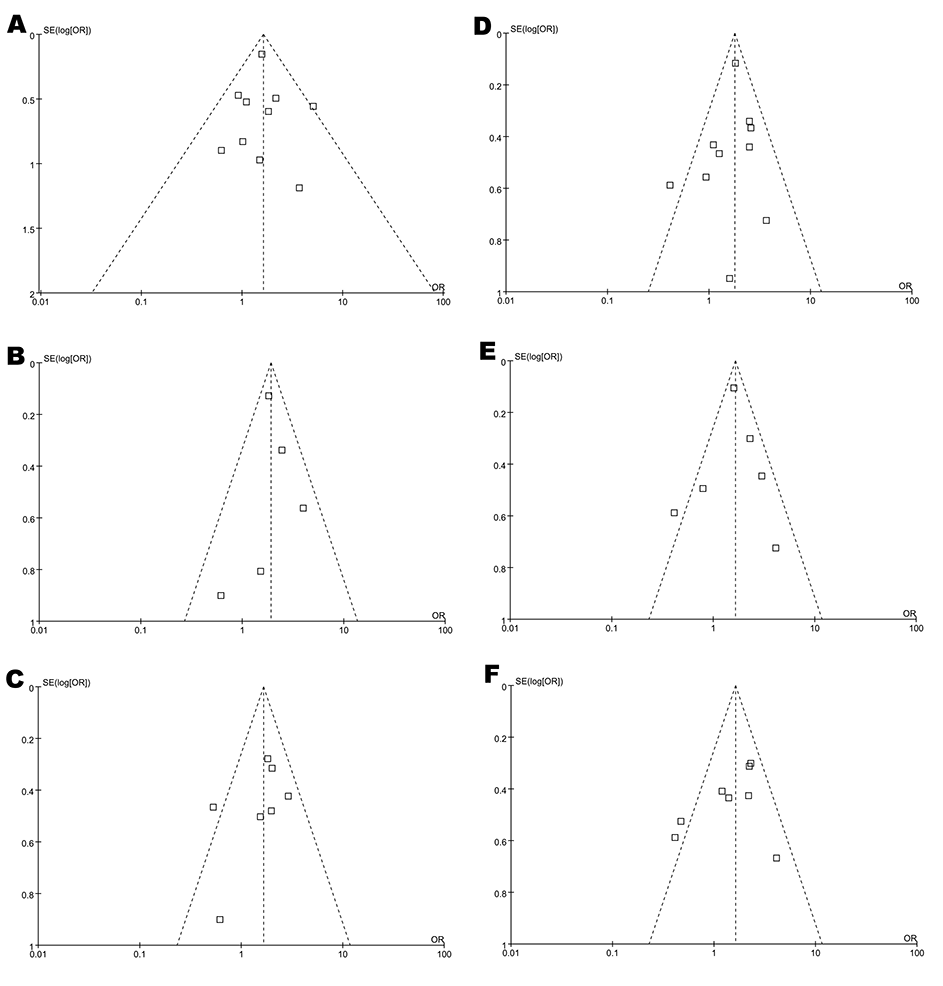

Supplement: S5 Fig — (A) 1-year patient survival, (B) 3-year patient survival, and (C) 5-year patient survival between WLT and TVLT; (D) 1-year graft survival, (E) 3-year graft survival, and (F) 5-year graft survival between WLT and TVLT. (TIF) [file pone.0138202.s006.tif]
