# Supplementary material for: Outcomes of Technical Variant Liver Transplantation versus Whole Liver Transplantation for Pediatric Patients: A Meta-Analysis
Source: PLoS One. 2015 Sep 14;10(9):e0138202. doi: 10.1371/journal.pone.0138202 (PMC4569420; doi:10.1371/journal.pone.0138202)
Supplement: S1 Text — (DOCX) [file pone.0138202.s007.docx]

**List of full-text excluded articles with reasons**

Full-text articles excluded, with reasons (n=31)

Not comparison between WLT and TVLT (n=7);

Lack of survival data (n=14)

Patients included adult (n=3)

Others (n=7)
